# Healthcare Utilization and Outcomes in Atrial Fibrillation Patients Treated by Drug Therapy versus a Catheter Ablation Strategy—A Middle European Propensity Score Matched Cohort Study

**DOI:** 10.3390/jcdd9120451

**Published:** 2022-12-10

**Authors:** Martin Martinek, Harry J. G. M. Crijns, Barbara A. B. Essers, Rene Wiesinger, Gerald Pruckner

**Affiliations:** 1 Internal Medicine II with Cardiology, Angiology, and Intensive Care Medicine, Ordensklinikum Linz Elisabethinen, 4020 Linz, Austria; 2 CARIM—Cardiovascular Research Institute Maastricht, Maastricht University, 6200 MD Maastricht, The Netherlands; 3 Institute of Health Economics, Johannes Kepler University Linz, 4020 Linz, Austria; 4 Christian Doppler Laboratory for Aging, Health, and the Labor Market, 4020 Linz, Austria

**Keywords:** atrial fibrillation, ablation, healthcare utilization, outcomes, mortality, labour market

## Abstract

Background and Aims: Atrial fibrillation (AF) is the most prevalent arrhythmia, associated with increased mortality and morbidity and causing relevant costs. Treatment options consist of catheter ablation (PVI) and rate or rhythm control drugs (non-PVI). Methods: We analyze inpatient and outpatient data from the Upper Austrian Health Insurance Fund. Data of patients with a first hospitalization for AF in the years 2005 to 2018 were examined, using propensity score matching (PSM) including all CHA_2_DS_2_-VASc variables and working “collar”. Results: Out of 21,791 AF patients, PSM identified 1013 well-matching pairs (PVI and non-PVI). Over a ten-year period, the PVI treatment strategy group reveals significantly higher inpatient and outpatient expenditures (€2200/year). Positive economic effects can be demonstrated by a 5.1 percentage points (pp) higher employment rate and fewer retirements (7.6pp). Of utmost important is the 5.8pp all-cause mortality reduction over 10 years in the PVI treatment strategy. Conclusions: A PVI based treatment strategy results in higher healthcare expenditures vs. drug therapy alone. Most of these higher costs were caused by the PVI procedures during this period. Thus, more effective and efficient methods are needed to further reduce costs for the intervention and prevent repeat procedures. The benefit of a PVI treatment strategy is seen in higher employment rates, which are crucial from a societal perspective and should be a strong argument for caregivers. We show a significant reduction in all-cause mortality, which we partly attribute to the PVI procedure itself, to a stricter risk factor assessment and treatment, and a tighter medical adherence.

## 1. Introduction

Atrial Fibrillation (AF) is the most common heart rhythm disorder in adult patients worldwide with a current estimated prevalence of 2–4% [1]. The 2020 European Society of Cardiology (ESC) Guidelines for the diagnosis and management of atrial fibrillation expect a 2.3-fold rise over the next decade due to the extended longevity in the general population and intensified search for undiagnosed AF. One in three Europeans at an index age of 55 will develop AF in their lifetime which poses relevant pressure on our health care systems [1]. AF-related outcomes include a 1.5–3.5-fold increase in mortality, stroke (20–30% of all ischemic strokes are due to AF), heart failure (in 20–30% of AF patients), cognitive decline and dementia (hazard ratio 1.4–1.6, irrespective if stroke). AF patients show impaired quality of life in more than 60%, thus leading to medical or ablation treatment and the annual hospitalization rate is as high as 10 to 40%, causing relevant costs in our health care budgets [1].

In 2020, our study group published an analysis of actual health care expenditure after PVI, showing significant cost-savings on post-interventional inpatient and outpatient expenditures and a reduction in days of sick leave in a PVI-population [2]. This new long-term analysis of an extended Upper Austrian cohort tried to broaden the scope by comparing healthcare expenditure, outcomes and mortality of drug therapy (non-PVI) and a PVI based treatment strategy.

In recent years progressive evidence has been accumulated that first-line PVI procedures may significantly improve outcomes using different ablation techniques [3,4]. Our study population of PVI patients mainly consists of second-line PVI, in earlier years correlating to former AF Guidelines, and may only have included first-line patient in recent years. The treatment strategies within the study may thus not represent the latest standards where early rhythm control has been shown to be superior [5].

The study is part of a PhD project at CARIM in conjunction with the DAS-CAM program (www.dascam.org, accessed on 2 February 2019) of the University of Maastricht, Maastricht University Medical Center, European Heart Academy, and the Johannes Kepler University Linz (JKU).

## 2. Methods

We identified all patients who were first hospitalized with paroxysmal or persistent AF as their main diagnosis in the years 2005 to 2018 with the respective ICD-10 (International Statistical Classification of Diseases and Related Health Problems) diagnoses (I48.*) and who were insured with the Upper Austrian Health Insurance Fund (OÖGKK). PVI patients are identified by the medical treatment group (MEL-codes: in German, Medizinische Einzelleistungen) 6546 (2005–2007), 6547 (2008), and DE060 (from 2009 on). The OÖGKK provides inpatient and outpatient health care data for more than 1.8 million employees and their dependents, representing more than 75% of the provincial population.

The final dataset includes 21,791 patients identified by their first hospitalization for AF (fhfAF) between Q1/2005 and Q4/2018. Of these, 1624 (7.5%) were treated with at least one PVI (1222 had one PVI, 328 had two, and 66 had three procedures), the rest received other treatments. Without patient matching, we observe significant differences in health care expenditures, and demographic and socio-economic characteristics between PVI patients and non-PVI patients (Table S1 Supplementary). PVI patients are substantially younger, predominantly male, and show fewer comorbidities as well as lower mortality rates after their fhfAF. To cope with these differences, we introduce a propensity score matching procedure.

The data set provides all-cause mortality for all patients whereas a further differentiation of the mode of death is difficult. Hence, we proceed as follows: If an individual was hospitalized in the quarter of their death, we assume they died in hospital. This holds true for approximately 70% of deaths. For these individuals, we are able to identify a diagnosis which is the base for the cause of death. We differentiate between three categories of hospital deaths: cardiovascular deaths (ICD 10 Code I*), neoplasm deaths (ICD 10 Codes C00*–D49*), and other deaths.

### 2.1. Catheter Ablation and Drug Therapy

PVI patients in our cohort were mainly treated by radiofrequency CA (RFCA) as cryoballoon technology entered the market mainly after 2010. In later years, the proportion of cryoballoon treated patients increased as this has become the main technology in one of the three Upper Austrian ablation centers. The ratio of RFCA versus cryoballoon in our patient cohort is at a rough estimate about two-thirds to one-third. More sophisticated RFCA catheters with force-management entered the market around 2011 and have been the leading technology as of 2012 with additional technological improvements throughout the years.

ESC guidelines up until 2018 recommended a rhythm control strategy in patients still symptomatic on proper rate-control therapy [6]. Both AAD therapy (Class IA indication) and PVI (Class IIaB indication) were possible as first-line rhythm-control strategies. Still, considering older ESC guidelines in the years from 2005, most PVI patients in the analyzed cohort are treated second line after failed AAD. Thus, many patients undergoing PVI are treated by both strategies for some time during the study period. Additionally, AAD therapy may also be used as adjunctive therapy for some months after PVI or in PVI patients who do not fully respond to ablation. We analyze AAD use by ATC codes (Anatomical Therapeutic Chemical Classification of the European Medicines Agency) and focus on differences in usage over time and changes after PVI, respectively.

Both AAD and rate-control therapy is possible in the non-PVI comparator group. As a robustness check, we also analyzed a PSM cohort including AAD treated patients only in the control group. The results are basically unchanged. However, we lose 200 patients in the analysis with no match and overall, the matching is inferior with respect to propensity scores.

### 2.2. Institutional Setting and Data

The Austrian “Bismarckian-type” health care system provides universal access to medical services for the whole population. Up until 2018 nine provincial health insurance funds offer mandatory health insurance for employees and their dependents, and most unemployed individuals. Membership in the regional health insurance funds cannot be chosen freely—it is determined by the individual’s place of residence. Self-employed persons and state employees are insured via alternative health insurance institutions that are not part of our database. All health care expenses in the inpatient and outpatient sector, including those for medication, are covered by the health insurance. Patients pay a prescription charge and a small deductible per day of hospital treatment.

### 2.3. Statistical Analysis and Propensity Score Matching

Continuous variables are presented as means with their corresponding confidence intervals. Ordinal parameters are depicted as counts, percentages, or percentage points (pp). The analysis of empirical data is based on regression analysis and Student’s *t*-tests for paired samples. Statistical significance is presented at the 1% level (highly significant, *p* < 0.01), the 5% level (significant, *p* < 0.05), and the 10% level (a trend towards significance, *p* < 0.1). The statistical analysis was performed using STATA software (StataCorp LLC, College Station, TX, USA).

We use propensity score matching (PSM) to provide a well-balanced non-PVI comparison group. Following an event study design, time has been normalized such that a value of zero marks the quarter of the fhfAF and negative (positive) values denote quarters before (after) this hospitalization. Beyond matching for age and gender, we use all other parameters of the CHA_2_DS_2_-VASc Score, renal failure, and hyperlipidemia, as well as blue or white collar, to implement the socio-economic status which might influence health care accessibility. The inclusion of the latter variable implies that we analyze individuals only who were employed at some point eight years before the fhfAF. All matching parameters are calculated for the year before the fhfAF. For a better robustness concerning “collar” we checked for white- and blue-collar employment eight years before fhfAF.

After PSM we receive very well-matched groups Table 1 of 1013 PVI and 1013 non-PVI patients with a good observability of matching specifications (Figure S1 Supplementary), an extremely low difference in propensity scores (Figure S2 Supplementary), and well-balanced cohorts with less than 10% differences in the single matching parameters (Figure S3 Supplementary). Thus, the PSM cohort provides a robust basis for the analysis.

## 3. Results

The final sample includes 2026 PVI-treated or non-PVI-treated patients. A total of 748 individuals underwent a single PVI, 215 had two, 44 three, and 6 four ablation procedures. Patients in this cohort are rather young and mainly male. All matching variables are provided in Table 1. Significantly different outcome variables already before fhfAF were higher expenditures for outpatient care in the year before fhfAF (€140.20 in PVI vs. €123.71 in non-PVI, *p* = 0.0001), employment (76.8% in PVI vs. 73.0% in non-PVI, *p* = 0.038), and prescription probability for AAD including Betablockers (27.0% in PVI vs. 10.0% in non-PVI, *p* = 0.0001), and anticoagulants (29.2% in PVI vs. 22.3% in non-PVI, *p* = 0.0001) (Table S2 Supplementary).

**Figure 1:**
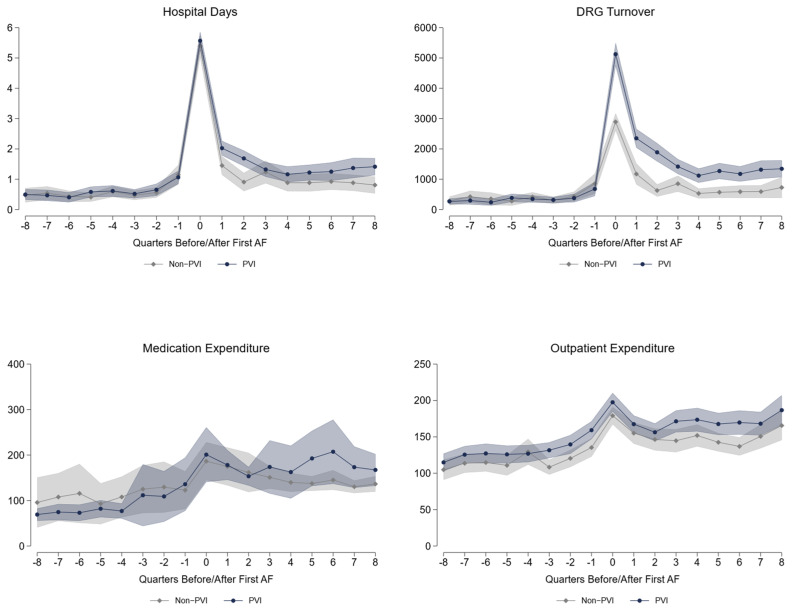
Short-term healthcare Expenditure and Hospital days.

For short-term comparison, we analyze eight quarters after the fhfAF Table 2. Both inpatient and outpatient expenditures are significantly higher in the PVI group, with a difference of €1025.59 per quarter or approximately €4100 per year, including medication Figure 1. Most of this difference arises in the first year after the fhfAF, as most PVI patients undergo their procedures during this period (28% within one quarter, 41% within two, 48% within three, and 54% within one year, additional 3% per quarter up to quarter 8, adding up to 66% within 2 years). In parallel, hospital days are also significantly higher in the PVI group with a difference of roughly 1.6 days per year, mainly gained in the first year Figure 1. We find a significant reduction in all-cause mortality of 1.6pp over the first two years. Whereas the number of cardioversions is significantly higher in the PVI group, pacemaker- or ICD-implantations do not differ. Employment remains significantly higher in the PVI group, mainly due to a lower retirement rate. On the other side, PVI patients have 4.5 more sick leave days per year than non-PVI patients. More coronary artery disease and hyperlipidemia is diagnosed in the PVI group over the first two years. Additionally, significantly more AAD including betablockers, anticoagulants, and lipid-lowering drugs are prescribed in the PVI group.

Health care utilization and medication expenditures are displayed in Euro per quarter, all other data show cumulative values over eight quarters after first hospitalization for atrial fibrillation. DRG—diagnosis-related groups; DRG turnover is the actual accounting calculated from the DRG points.

**Figure 2:**
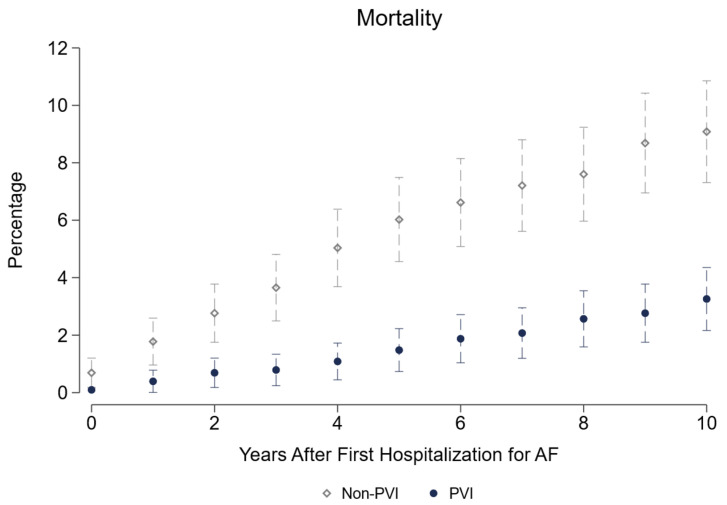
Long-term healthcare Expenditure and Hospital days.

**Figure 3:**
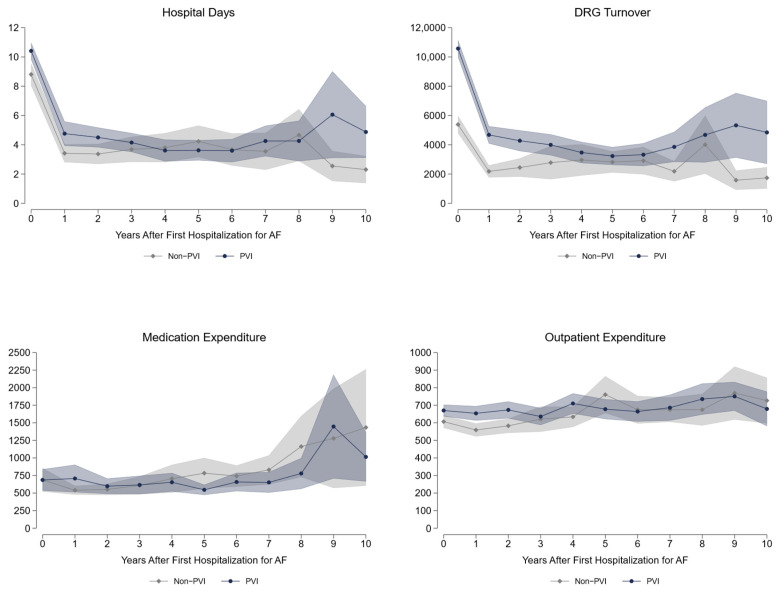
All-cause Mortality.

Long-term data up to 10 years after fhfAF reveal a significantly higher health care utilization in the PVI treatment strategy group with respect to hospital days, inpatient expenditures (diagnosis-related groups (DRG) turnover is the actual accounting calculated from the DRG points), and outpatient medical attendance Figure 2. As can be seen from Table 3, PVI patients roughly spend €2200 more per year than non-PVI patients, with most difference in inpatient costs. Their need for pacemaker implantation is significantly higher (+2.5pp) and the same holds true for cardioversion (+19pp). More coronary artery disease and hyperlipidemia with a trend to lower heart failure diagnosis is seen in PVI, whereas the probability of peripheral artery disease is higher in non-PVI patients. Significantly more AAD including betablockers, anticoagulants, and lipid-lowering drugs are prescribed in the PVI based treatment group with a higher prescription rate of antidiabetic drugs in the non-PVI group. Regarding labor market outcomes, we demonstrate a significantly higher employment rate (+5.1pp), due to reduced retirements (−7.6pp). On the contrary, sick leave days are roughly 3 days more per year for PVI patients. Of highest importance, all-cause mortality shows a 5.8pp reduction over 10 years in the PVI based treatment strategy. The largest share of this difference arises in the first 5 years Figure 3. Modes of death were not different in both groups, as can be seen by the absolute numbers presented in Table 4.

Health care utilization data are displayed per year; all other data show cumulative values over ten years. DRG—diagnosis-related groups; DRG turnover is the actual accounting calculated from the DRG points.

## 4. Discussion

### 4.1. Health Care Expenditures

To the best of our knowledge this is the first empirical analysis based on real inpatient and outpatient health care expenditures in AF patients comparing a PVI based strategy vs. drug therapy (non-PVI). We find an increase in health care expenditures of roughly €4100 per year in the short run, which levels down to €2200 per year over 10 years after fhfAF. As most PVI procedures are performed within two years after the first AF hospitalization, the short-term increase in expenditures mainly reflects costs for the ablation procedure itself, that is calculated with €9873 (MEL-coding DE060) in Austria. A second driver of expenditures in the PVI group is the higher cardioversion rate in both time periods and a higher rate of pacemaker implantation in the long run. The third component of higher expenditures in the PVI group is the increase in outpatient health care. The higher short-run expenditures for medication in PVI patients levels out in the long run. Concerning relevant medication for cardio-vascular disease, the prescription probability of anticoagulants, antiarrhythmic, and lipid-lowering drugs is significantly higher in the PVI-group. Both higher outpatient costs as well as a higher usage of these relevant medications could partly explain the lower mortality rate of the PVI group.

Other recent studies on the impact of PVI show that total AF costs were higher during the first year after ablation vs. before ablation obviously owing to the PVI procedures [2,7]. However, after 18-month follow-up post ablation AF-related costs per-patient-per-month were reduced, despite including costs from repeat ablation [7]. We reported similar effects in patients pre- and post-PVI in a previous study [2]. In the present study, health care expenditures in the PVI group are still higher in the long run, however we include all inpatient and outpatient expenses, not only AF-related components and additionally compare with drug treated patients. Gupta et al. report a 42% reduction in cardiovascular hospitalizations in a single-arm study of patients treated by PVI using radiofrequency ablation and the CLOSE protocol [8]. We also see a significant reduction in hospital days in our first single-arm study [2]. In the present study, PVI and non-PVI patients show a significant reduction of hospital days in the quarters after their fhfAF, and the effect is even more pronounced in the non-PVI group. Of note, our point zero is defined by the fhfAF and not by the date of the actual PVI intervention, which could explain part of the differences. Also, the actual time of PVI (early vs. late) may have a difference on further hospitalizations and outcome. Chun et al. report lower costs of cryoablation vs. radiofrequency ablation in the FIRE and ICE trial [9], whereas Murray et al. find higher costs in cryoablation but a more favorable cost per QALY vs. radiofrequency ablation [10]. In our study, we cannot differentiate between ablation methods as there is only one single MEL-code for PVI without further differentiation. It may be that a higher proportion of cryoablation would cause lower long-term expenditures in the PVI group. Chun et al. identify repeated ablations as the largest cost driver with a lower incidence of repeated procedures in cryoablation. They use payment rates for rehospitalizations within 1.5 years for their comparison, exclude the costs of the index ablation, and do not include expenditures for outpatient care or medication, so that a direct comparison to our data is not possible. Nevertheless, we observe repeated ablations in 26.1% of treated patients in our sample which apparently increases health care expenditures. Therefore, it is mandatory to further improve ablation technologies. In general, most ablation trials rely on short follow-up times. In contrast, we provide healthcare resource use data over a period of more than 10 years. Randomized controlled trials are defined by strict inclusion and exclusion criteria and might lack external validity. We focus on a real-world population treated with PVI or medical drugs at a certain point in time. This population represents everyday patients treated for AF better than patients included in trials.

### 4.2. Labour Market Outcomes

We find a 5pp higher employment rate in patients treated with a PVI strategy—mainly driven by the lower retirement rate—in the short- and long-run, whereas the treated individuals only have three more sick leave days per year over a period of 10 years, which does not result in substantial productivity loss. No other study in AF treatment has ever included labour market outcomes so far. This is surprising, as—from a health economic perspective—including employability and sick leave provides crucial information on potential benefit of an intervention from a broader societal perspective.

The benefit of a PVI based strategy resulting in significantly higher employment rates is crucial for the gross economic impact of a treatment strategy and should be a strong argument for caregivers to provide sufficient catheter ablation resources.

### 4.3. Mortality

All-cause mortality is of course the hardest and most important endpoint in medical interventions. We report a 0.8pp (0.6pp) reduction per year in the short (long) run, adding up to 5.8pp after ten years. Given that total numbers are small and only 70% of these patients died in-hospital, we cannot reliably differentiate between causes of death. Cardiovascular death was numerically reduced in the PVI group, but, to an even greater degree, neoplasm and other modes of death. The reduction in neoplasm and other hospital deaths may again be attributed to a more rigorous assessment and an overall tighter medical adherence in the PVI strategy. For all these reasons we speak of a “PVI based treatment strategy”, not only of “PVI” in this manuscript.

While a trend to lower heart failure diagnoses may be explained by the PVI itself, the higher rate of coronary artery disease and hyperlipidemia might reflect a stricter risk factor assessment in individuals undergoing PVI. One the one hand, this may again drive expenditures in the PVI group, but may be beneficial for mortality in the long run due to early therapeutic intervention. Additionally, despite PSM and same CHA_2_DS_2_-VASc risk factors much more patients were on anticoagulation in the PVI group before and during follow-up which could add to the mortality benefit even given the low stroke rates. Giving credit to the results of the EAST-AFNET^5^ study, the markedly higher amount of AAD use before and after fhfAF could add to the mortality benefit in the PVI group. With regards to medication, the significantly higher number of lipid-lowering drugs could have another positive effect on mortality in the PVI group.

Saliba et al. find a 43% relative risk reduction for mortality in a PSM model of a similar PVI population vs. drug-treated patients [11]. They report the main difference in stroke or transient ischemic attack (TIA) risk, which we do not see in our population. Mortality rates over a maximum follow-up of 11 years are similar to our population providing a similar time horizon in both the PVI group (4.66% vs. 3.3% in our study) and the non-PVI group (7.25% vs. 9.1% in our study). Two large randomized control trials CABANA [12] and EAST-AFNET [5] have analyzed mortality in AF populations. CABANA demonstrates a mortality benefit for PVI only in the “on-treatment” analysis. Similar to CABANA, many patients in our cohort may have crossed over from AAD therapy to PVI, which may be part of the explanation of the high percentages of AAD in the PVI group. Our PVI based strategy cohort might be very similar to the on-treatment arm of CABANA and show similar results. EAST-AFNET was stopped for efficacy after a median of 5.1 years of follow-up per patient and demonstrated a reduction in the composite endpoint of death from cardiovascular causes, stroke, or hospitalization with worsening of heart failure or acute coronary syndrome. Here, death from cardiovascular causes and stroke were significantly reduced by early rhythm control therapy. Patients included in EAST-AFNET would be spread into both study arms in our study with high percentages of AAD use in both the PVI and the non-PVI group. However, AAD use is still significantly higher in PVI patients over the time course. We cannot explicitly observe the effect of early rhythm control in our population, and we cannot claim that PVI patients were treated earlier than AAD-treated patients. Still, we know that 54% of PVI procedures were provided within the first year which would fit to the early rhythm control concept in the EAST-AFNET trial. The EAST-AFNET trial was criticized for a potential bias due to a closer follow-up in the early rhythm control group due to more frequent electrocardiograms and therefore a potentially higher medical attention. The same could be true in our PVI based group and explain part of the mortality benefit.

## 5. Limitations

The study has several limitations. This is a non-randomized observational cohort study comparing drug therapy vs. a PVI based strategy for AF treatment. Patients were included based on the respective ICD and MEL codes. Obviously, ICD-10 and MEL coding quality are crucial for identifying potential subjects. ICD codes for the first diagnosis and MEL coding for PVI seem to be highly reliable as they represent the basis for correct hospital invoicing. In contrast, secondary ICD diagnoses may be less reliable as they are not crucial for charging purposes. However, most diagnoses relevant for subsequent drug prescription are included in the hospital’s patient letter, for example, factors of the CHA_2_DS_2_-VASc-Score, chronic kidney disease, or hyperlipidemia, which we used for PSM. We cannot provide comparisons between paroxysmal or persistent AF, as the ICD code I48 was only separated after 2015 to I48.0 and I48.1.

In our study we cannot differentiate between different ablation techniques. We estimate that two thirds of the procedures were done using RF and one third using cryoablation. No other technologies such as the laser-balloon were available. Different costs of RF vs. cryoablation were not evaluated in the current study as the Austrian system does not differentiate methods for the hospital compensation.

PSM always faces the problem of potential confounders that are not within the analyzed parameters and not even known yet. Still, we included the most relevant known parameters for morbidity and mortality in cardiovascular disease as well as “collar” as socio-economic parameter influencing medical adherence in the PSM to provide a fair comparison. Only patients with hospitalizations for the main diagnosis of AF were analyzed to prevent including patients with AF as a complication of other medical issue, not necessarily represented in the PSM, which may then affect treatment decision and long-term outcomes.

A retrospective PSM cohort study never can show causal effects, but we try to elucidate potential relationships that have to be proven in randomized clinical trials. Numerous reasons exist why patients are being treated by drugs or undergo a PVI. Even if we try to provide a reliable comparison between drug and PVI therapy, we cannot fully compensate for the potential selection bias of patients introduced by treating physicians in an all-comer AF cohort.

By including the “collar”, we create a rather young patient group that is another five years younger than the overall PVI patients in our cohort. Nevertheless, it was the best way to include socio-economic status in the PSM. Females are underrepresented in our PSM, so we cannot generalize the results to the whole population. Of all PVI patients included in this study, only 31.6% are female, showing the fact that PVI is mainly provided in male patients, also seen in other studies [12].

## 6. Conclusions

Analyzing a cohort of 2026 PSM patients out of 21,791 patients identified by their fhfAF in the years 2005 to 2018, a PVI based treatment strategy results in higher healthcare expenditures vs. drug therapy alone (non-PVI) over short and long-term periods. Most of this cost excess is a result of the PVI procedures. Thus, more effective and efficient methods are needed to further reduce costs for the intervention, but also to prevent repeat procedures, and make AAD use after PVI obsolete. Next to the investment in costs, our results show a significant reduction in all-cause mortality in PVI patients which partly can be seen as a direct health benefit of the PVI procedure itself but potentially also an indirect result of a stricter risk factor assessment and treatment, a rigorous medical work-up and a tighter medical care in patients undergoing a PVI based strategy. We cannot claim a causal effect of PVI on mortality as this has not been shown in RCTs.

The benefit of a PVI strategy is seen in significantly higher employment rates, which is crucial for the gross economic impact and should be a strong argument for caregivers to provide catheter ablation resources. We can show a significant reduction in all-cause mortality in patients treated with a PVI based strategy, which we partly attribute to the PVI procedure itself, to a stricter risk factor assessment and treatment, a rigorous medical work-up, and a tighter medical care in patients undergoing PVI.

## Figures and Tables

**Table 1:** Patient characteristics and risk factors used for propensity score matching.

	Ø Non-PVI	Ø PVI	Diff.	*p*-Value	95% CI
**Patient Characteristics**					
Female	0.208	0.202	−0.006	0.742	[−0.041, 0.029]
Age at First AF	53.170	53.619	0.449	0.271	[−0.351, 1.249]
Age at First AF < 55	0.498	0.481	−0.017	0.450	[−0.060, 0.027]
Age at First AF 55–65	0.444	0.461	0.017	0.448	[−0.027, 0.060]
Age at First AF > 65	0.058	0.058	0.000	1.000	[−0.020, 0.020]
Blue Collar	0.393	0.397	0.004	0.875	[−0.046, 0.054]
**Risk Factor Diagnoses**					
Heart Failure	0.011	0.016	0.005	0.333	[−0.005, 0.015]
Hypertension	0.045	0.065	0.020 *	0.052	[−0.000, 0.040]
Diabetes	0.007	0.016	0.009 *	0.059	[−0.000, 0.018]
TIA/Stroke	0.006	0.010	0.004	0.316	[−0.004, 0.012]
Coronary Heart Disease	0.034	0.048	0.015 *	0.093	[−0.002, 0.032]
Peripheral Artery Disease	0.002	0.001	−0.001	0.564	[−0.004, 0.002]
Hyperlipidemia	0.022	0.036	0.014 *	0.062	[−0.001, 0.028]
Renal Failure	0.001	0.002	0.001	0.564	[−0.002, 0.004]

Note—* *p* < 0.1. Standard errors are corrected for heteroscedasticity.

**Table 2:** Short-term Outcomes eight quarters after first hospitalization for atrial fibrillation.

	Ø Non-PVI	Ø PVI	Diff.	*p*-Value	95% CI
**Health Care Utilisation**					
Hospital Days	1.580	1.981	0.401 ***	0.000	[0.256, 0.547]
DRG Points	773.238	1518.317	745.079 ***	0.000	[642.961, 847.196]
DRG Turnover	1000.059	1979.464	979.406 ***	0.000	[846.268, 1112.543]
Drug Expenditure	153.151	179.010	25.859 **	0.011	[5.888, 45.829]
Outpatient Medical Care	152.991	173.318	20.327 ***	0.000	[13.346, 27.308]
**Mortality**					
All-Cause Mortality	0.020	0.004	−0.016 ***	0.001	[−0.025, −0.006]
In-Hospital Death	0.158	0.051	−0.107 **	0.042	[−0.209, −0.004]
Cardiovascular Death	0.138	0.074	−0.064	0.338	[−0.197, 0.068]
Neoplasm Death	0.031	0.037	0.006	0.884	[−0.078, 0.091]
Other Death Cause	0.062	0.000	−0.062 **	0.044	[−0.121, −0.002]
**Pacemakers and Cardioversion**					
Pacemaker	0.015	0.022	0.007	0.246	[−0.005, 0.019]
Implantable Cardioverter-Defi.	0.013	0.008	−0.005	0.273	[−0.014, 0.004]
Cardioversion	0.273	0.384	0.111 ***	0.000	[0.070, 0.151]
**Labour Market Outcomes**					
Employed	0.700	0.753	0.053 ***	0.008	[0.014, 0.092]
Unemployed	0.106	0.096	−0.011	0.427	[−0.037, 0.016]
Retired	0.390	0.330	−0.060 ***	0.005	[−0.103, −0.018]
Blue Collar	0.388	0.402	0.014	0.599	[−0.038, 0.065]
Sick Leave Days	5.865	6.991	1.125 ***	0.005	[0.342, 1.909]
**Risk Factor Diagnoses**					
Heart Failure	0.077	0.060	−0.017	0.135	[−0.039, 0.005]
Hypertension	0.246	0.243	−0.003	0.877	[−0.040, 0.034]
Diabetes	0.057	0.048	−0.009	0.372	[−0.028, 0.011]
TIA/Stroke	0.026	0.022	−0.004	0.559	[−0.017, 0.009]
Coronary Heart Disease	0.114	0.165	0.051 ***	0.001	[0.021, 0.081]
Peripheral Artery Disease	0.011	0.003	−0.008 **	0.032	[−0.015, −0.001]
Hyperlipidemia	0.137	0.219	0.082 ***	0.000	[0.049, 0.115]
Renal Failure	0.007	0.007	−0.000	1.000	[−0.007, 0.007]
Dementia	0.001	0.000	−0.001	0.317	[−0.003, 0.001]
**Relevant Medication**					
*Expenditures*					
Antithrombotic (B01*)	31.300	46.202	14.902 ***	0.000	[12.087, 17.718]
Antiarrhythmic (C01*)	12.690	20.218	7.529 ***	0.000	[6.238, 8.819]
Antihypertensive (C02*)	3.218	1.159	−2.059 *	0.077	[−4.344, 0.225]
Antidiabetic (A10*)	6.297	4.098	−2.198 ***	0.000	[−3.208, −1.188]
Lipid-Lowering Drugs (C10*)	7.439	9.189	1.750 ***	0.002	[0.627, 2.873]
*Prescription Probability*					
Antithrombotic (B01*)	0.577	0.803	0.226 ***	0.000	[0.187, 0.265]
Antiarrhythmic (C01*)	0.397	0.660	0.264 ***	0.000	[0.222, 0.306]
Antihypertensive (C02*)	0.067	0.052	−0.015	0.160	[−0.035, 0.006]
Antidiabetic (A10*)	0.082	0.060	−0.022 *	0.057	[−0.044, 0.001]
Lipid-Lowering Drugs (C10*)	0.272	0.336	0.063 ***	0.002	[0.023, 0.103]
**Drugs by ATC Chapter**					
Alimentary Tract	14.013	10.991	−3.022 ***	0.000	[−4.306, −1.739]
Blood	37.141	46.557	9.416 ***	0.005	[2.833, 15.998]
Cardiovascular System	51.418	55.555	4.137 **	0.016	[0.774, 7.501]
Nervous System	11.443	5.845	−5.597 ***	0.000	[−6.996, −4.198]
Respiratory System	7.164	6.179	−0.984 *	0.082	[−2.093, 0.124]

Note—*** *p* < 0.01, ** *p* < 0.05, * *p* < 0.1. Standard errors are corrected for heteroscedasticity.

**Table 3:** Long-term Outcomes ten years after first hospitalization for atrial fibrillation.

	Ø Non-PVI	Ø PVI	Diff.	*p*-Value	95% CI
**Health Care Utilisation**					
Hospital Days	4.592	5.421	0.829 ***	0.000	[0.430, 1.228]
DRG Points	2381.206	4014.588	1633.382 ***	0.000	[1350.966, 1915.798]
DRG Turnover	3121.271	5297.225	2175.954 ***	0.000	[1806.870, 2545.039]
Drug Expenditure	706.593	682.043	−24.550	0.550	[−105.048, 55.948]
Outpatient Medical Care	630.563	674.044	43.480 ***	0.001	[18.350, 68.611]
**Mortality**					
All-Cause Mortality	0.091	0.033	−0.058 ***	0.000	[−0.079, −0.037]
In-Hospital Death	0.674	0.564	−0.110	0.244	[−0.295, 0.075]
Cardiovascular Death	0.344	0.545	0.202	0.105	[−0.043, 0.446]
Neoplasm Death	0.359	0.318	−0.041	0.727	[−0.275, 0.192]
Other Death Cause	0.312	0.182	−0.131	0.203	[−0.333, 0.072]
**Pacemakers and Cardioversion**					
Pacemaker	0.028	0.052	0.025 ***	0.005	[0.008, 0.042]
Implantable Cardioverter-Defi.	0.022	0.014	−0.008	0.179	[−0.019, 0.004]
Cardioversion	0.333	0.522	0.190 ***	0.000	[0.147, 0.232]
**Labour Market Outcomes**					
Employed	0.708	0.759	0.051 ***	0.009	[0.013, 0.090]
Unemployed	0.150	0.139	−0.011	0.496	[−0.042, 0.020]
Retired	0.568	0.492	−0.076 ***	0.001	[−0.119, −0.032]
Blue Collar	0.408	0.408	0.001	0.982	[−0.051, 0.052]
Sick Leave Days	14.881	17.895	3.014 ***	0.000	[1.333, 4.695]
**Risk Factor Diagnoses**					
Heart Failure	0.125	0.102	−0.024 *	0.093	[−0.051, 0.004]
Hypertension	0.314	0.309	−0.005	0.811	[−0.045, 0.035]
Diabetes	0.088	0.075	−0.013	0.291	[−0.037, 0.011]
TIA/Stroke	0.054	0.044	−0.010	0.305	[−0.029, 0.009]
Coronary Heart Disease	0.192	0.256	0.064 ***	0.001	[0.028, 0.100]
Peripheral Artery Disease	0.026	0.011	−0.015 **	0.013	[−0.026, −0.003]
Hyperlipidemia	0.169	0.280	0.112 ***	0.000	[0.075, 0.148]
Renal Failure	0.018	0.016	−0.002	0.730	[−0.013, 0.009]
**Relevant Medication**					
*Expenditure*					
Antithrombotic (B01*)	117.731	157.073	39.342 ***	0.000	[28.156, 50.528]
Antiarrhythmic (C01*)	45.969	67.853	21.884 ***	0.000	[16.418, 27.350]
Antihypertensive (C02*)	16.376	7.759	−8.618	0.226	[−22.579, 5.343]
Antidiabetic (A10*)	30.834	23.352	−7.482 ***	0.005	[−12.750, −2.214]
Lipid-Lowering Drugs (C10*)	31.896	36.893	4.997 **	0.033	[0.403, 9.591]
*Prescription Probability*					
Antithrombotic (B01*)	0.716	0.954	0.238 ***	0.000	[0.207, 0.269]
Antiarrhythmic (C01*)	0.497	0.777	0.280 ***	0.000	[0.240, 0.320]
Antihypertensive (C02*)	0.110	0.100	−0.010	0.468	[−0.037, 0.017]
Antidiabetic (A10*)	0.134	0.099	−0.036 **	0.013	[−0.063, −0.008]
Lipid-Lowering Drugs (C10*)	0.399	0.471	0.072 ***	0.001	[0.029, 0.115]
**Drugs by ATC Chapter**					
Alimentary Tract	57.772	48.334	−9.438 ***	0.002	[−15.439, −3.437]
Blood	134.807	159.725	24.918 *	0.054	[−0.403, 50.238]
Cardiovascular System	202.487	210.753	8.266	0.354	[−9.230, 25.762]
Nervous System	54.420	24.676	−29.743 ***	0.000	[−36.594, −22.893]
Respiratory System	36.154	27.265	−8.889 ***	0.002	[−14.476, −3.303]

Note—*** *p* < 0.01, ** *p* < 0.05, * *p* < 0.1. Standard errors are corrected for heteroscedasticity.

**Table 4:** Number of Deaths.

	# Non-PVI	# PVI	Total Number
In-Hospital Deaths	64	22	86
Cardiovascular Deaths	22	12	34
Neoplasm Deaths	23	7	30
Other In-Hospital Deaths	20	4	24
Deaths Outside Hospital	28	11	39
**Total Deaths**	**92**	**33**	**125**

## Data Availability

Not applicable.

## Supplementary Materials

The following supporting information can be downloaded at: https://www.mdpi.com/article/10.3390/jcdd9120451/s1, Table S1: Comparison of all non-PVI and PVI patients in the database without matching. Gross data of all Atrial Fibrillation patients in the data base (medically treated by rate or rhythm control versus PVI) showing significant differences demographics, cardiovascular risk factors, in health care expenditure and socio-economic characteristics. DRG-diagnosis-related groups; DRG turnover is the actual accounting calculated from the DRG points. Table S2: Patient characteristics after propensity score matching; DRG-diagnosis-related groups. Figure S1: Observability of Matching Specifications; Number of observations of the propensity score parameters. Figure S2: Difference in Propensity Scores of Matching Specifications; abs = absolute difference in propensity scores for each matching pair. Figure S3: Balancing Check—Standardized % bias; The standardized % bias is the difference in means between non-PVI and PVI patients as a percentage of the average standard deviation of both groups.

## Abbreviations

AAD	Antiarrhythmic drug therapy
AF	Atrial fibrillation
DRG	DRG-diagnosis-related groups
ESC	European Society of Cardiology
fhfAF	First hospitalization for AF
MEL	Medical procedure groups–in German: Medizinische Einzelleistungen
Non-PVI	Rhythm or rate control drug therapy
pp	Percentage points (absolute difference between two percentages)
PSM	Propensity score matching
PVI	Pulmonary vein isolation/Catheter ablation therapy
RFCA	Radiofrequency catheter ablation
TIA	Transient Ischemic Attack
