# Supplementary material for: Healthcare Utilization and Outcomes in Atrial Fibrillation Patients Treated by Drug Therapy versus a Catheter Ablation Strategy—A Middle European Propensity Score Matched Cohort Study
Source: J Cardiovasc Dev Dis. 2022 Dec 10;9(12):451. doi: 10.3390/jcdd9120451 (PMC9781316; doi:10.3390/jcdd9120451)
Supplement: Supplementary file 1 [file jcdd-09-00451-s001.zip › jcdd-2046861-supplementary.pdf]

Table S1. suppl. – Comparison of all non-PVI and PVI patients in the database without matching.

	Ø Non - PVI	Ø PVI	Diff.	95% CI		Sign.
**Patient Characteristics**						
Female	0.520	0.316	-0.204	-0.229	-0.179	***
Age†	71.557	58.267	-13.290	-13.926	-12.654	***
Age < 55†	0.095	0.337	0.241	0.226	0.257	***
Age 55 -- 65†	0.176	0.395	0.219	0.199	0.239	***
Age > 65†	0.728	0.268	-0.460	-0.483	-0.438	***
Deceased until 2018	0.314	0.081	-0.233	-0.256	-0.210	***
**Labour Market Outcomes**						
Employed†	0.164	0.531	0.366	0.346	0.387	***
Unemployed†	0.016	0.030	0.014	0.007	0.021	***
Retired†	0.838	0.466	-0.372	-0.392	-0.351	***
**Risk Factor Diagnoses**						
Heart Failure	0.259	0.142	-0.118	-0.139	-0.096	***
Hypertension	0.148	0.123	-0.026	-0.044	-0.008	***
Diabetes	0.064	0.034	-0.030	-0.042	-0.018	***
TIA/Stroke	0.154	0.091	-0.064	-0.082	-0.046	***
Coronary Heart Disease	0.237	0.280	0.044	0.022	0.065	***
Peripheral Artery Disease	0.076	0.033	-0.043	-0.056	-0.030	***
Hyperlipidemia	0.003	0.011	0.008	0.005	0.011	***
Renal Failure	0.067	0.023	-0.045	-0.057	-0.032	***
Dementia	0.044	0.009	-0.036	-0.046	-0.026	***
**Four Quarters Before First AF**						
Hospital Days	1.683	0.907	-0.776	-0.923	-0.630	***
DRG Points	750.880	429.658	-321.222	-398.021	-244.424	***
DRG Turnover	976.135	549.943	-426.192	-526.452	-325.932	***
Drug Expenditure	218.872	137.542	-81.330	-108.562	-54.099	***
Outpatient Medical Care	172.055	160.098	-11.957	-18.593	-5.321	***
Sick Leave Days	0.840	2.600	1.761	1.492	2.029	***
**Quarter of First AF**						
Hospital Days	8.538	6.311	-2.227	-2.668	-1.787	***
DRG Points	3,195.315	4,268.696	1,073.381	836.560	1,310.201	***
DRG Turnover	4,159.938	5,503.132	1,343.193	1,033.377	1,653.010	***
Drug Expenditure	308.309	222.561	-85.747	-138.807	-32.687	***
Outpatient Medical Care	226.005	212.559	-13.446	-26.165	-0.728	**
Sick Leave Days	2.116	7.786	5.670	4.996	6.343	***
**Four Quarters After First AF**						
Hospital Days	2.620	1.910	-0.710	-0.891	-0.530	***
DRG Points	1,103.234	1,417.350	314.116	222.959	405.273	***
DRG Turnover	1,441.653	1,836.759	395.105	275.492	514.719	***
Drug Expenditure	301.364	206.549	-94.815	-127.811	-61.819	***
Outpatient Medical Care	198.874	182.281	-16.593	-23.416	-9.770	***
Sick Leave Days	1.448	5.189	3.741	3.340	4.141	***

Note - Gross data of all Atrial Fibrillation (AF) patients in the data base (medically treated by rate or rhythm control versus PVI) showing significant differences in demographics, cardiovascular risk factors, in health care expenditure and socio-economic characteristics. DRG – diagnosis-related groups; DRG turnover is the actual accounting calculated from the DRG points. *** $p < 0.01$. † - measured at first AF

Table S2. suppl. – Patient characteristics after propensity score matching.

	Ø Non - PVI	Ø PVI	Diff.	p-value	95% CI
**Health Care Utilisation**					
Hospital Days	0.709	0.725	0.017	0.824	[-0.130, 0.163]
DRG Points	397.792	340.071	-57.721	0.273	[-160.955, 45.513]
DRG Turnover	514.829	439.525	-75.304	0.273	[-209.993, 59.386]
Drug Expenditure	121.718	109.760	-11.958	0.521	[-48.473, 24.557]
Outpatient Medical Care	123.710	140.203	16.493***	0.000	[7.632, 25.354]
**Pacemakers and Cardioversion**					
Pacemaker	0.001	0.002	0.001	0.564	[-0.002, 0.004]
Implantable Cardioverter-Defi.	0.002	0.000	-0.002	0.157	[-0.005, 0.001]
Cardioversion	0.007	0.019	0.012**	0.018	[0.002, 0.022]
**Labour Market Outcomes**					
Employed	0.730	0.768	0.038**	0.046	[0.001, 0.076]
Unemployed	0.068	0.064	-0.004	0.721	[-0.026, 0.018]
Retired	0.288	0.255	-0.034*	0.089	[-0.072, 0.005]
Blue Collar	0.393	0.397	0.004	0.875	[-0.046, 0.054]
Sick Leave Days	3.928	3.950	0.022	0.964	[-0.929, 0.973]
**Relevant Medication**					
Expenditures					
Antithrombotic (B01*)	8.497	13.576	5.078***	0.000	[2.780, 7.377]
Antiarrhythmic (C01*)	2.391	9.638	7.247***	0.000	[6.083, 8.411]
Antihypertensive (C02*)	8.160	0.976	-7.184*	0.062	[-14.715, 0.347]
Antidiabetic (A10*)	4.434	2.503	-1.930***	0.001	[-3.061, -0.800]
Lipid-Lowering Drugs (C10*)	4.239	5.352	1.113*	0.066	[-0.072, 2.297]
Prescription Probability					
Antithrombotic (B01*)	0.223	0.292	0.069***	0.000	[0.031, 0.107]
Antiarrhythmic (C01*)	0.100	0.270	0.171***	0.000	[0.138, 0.204]
Antihypertensive (C02*)	0.047	0.031	-0.017*	0.051	[-0.034, 0.000]
Antidiabetic (A10*)	0.050	0.042	-0.008	0.398	[-0.026, 0.010]
Lipid-Lowering Drugs (C10*)	0.126	0.142	0.016	0.297	[-0.014, 0.046]
**Drugs by ATC Chapter**					
Alimentary Tract	10.731	7.977	-2.754***	0.000	[-4.287, -1.220]
Blood	30.746	13.733	-17.014*	0.091	[-36.750, 2.723]
Cardiovascular System	34.186	33.881	-0.305	0.941	[-8.339, 7.729]
Nervous System	9.152	4.037	-5.114***	0.000	[-6.962, -3.266]
Respiratory System	6.258	5.369	-0.889	0.261	[-2.441, 0.663]

Note – DRG – diagnosis related groups. *** $p < 0.01$, ** $p < 0.05$, * $p < 0.1$. Standard errors are corrected for heteroscedasticity.

Figure S1. suppl. – Observability of Matching Specifications. Number of observations of the propensity score parameters.

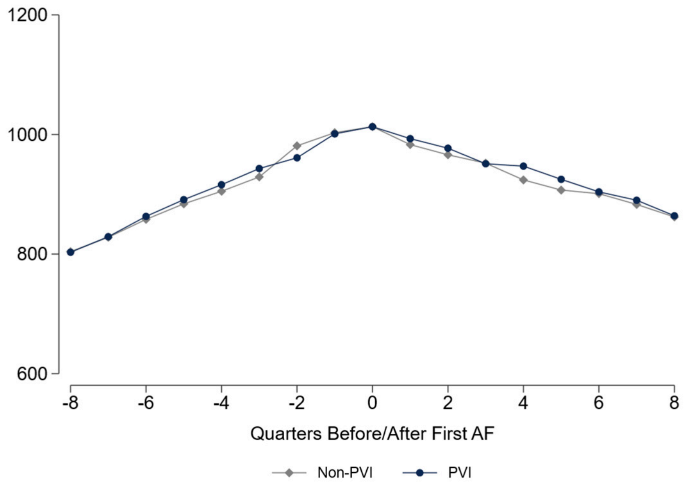

Figure S2. suppl. – Difference in Propensity Scores of Matching Specifications.

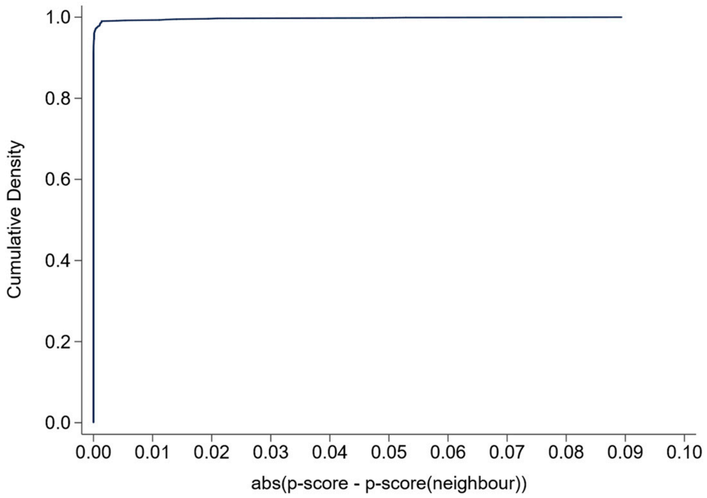

abs = absolute difference in propensity scores for each matching pair

Figure S3. suppl. – Balancing Check – Standardized % bias.

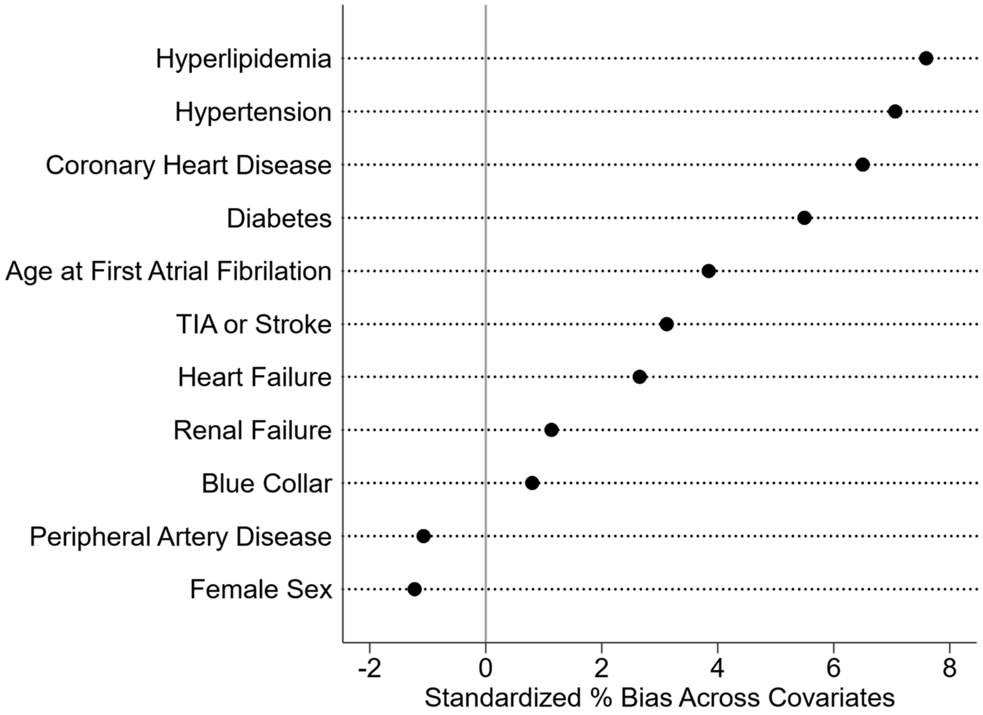

The standardized % bias is the difference in means between non-PVI and PVI patients as a percentage of the average standard deviation of both groups.
